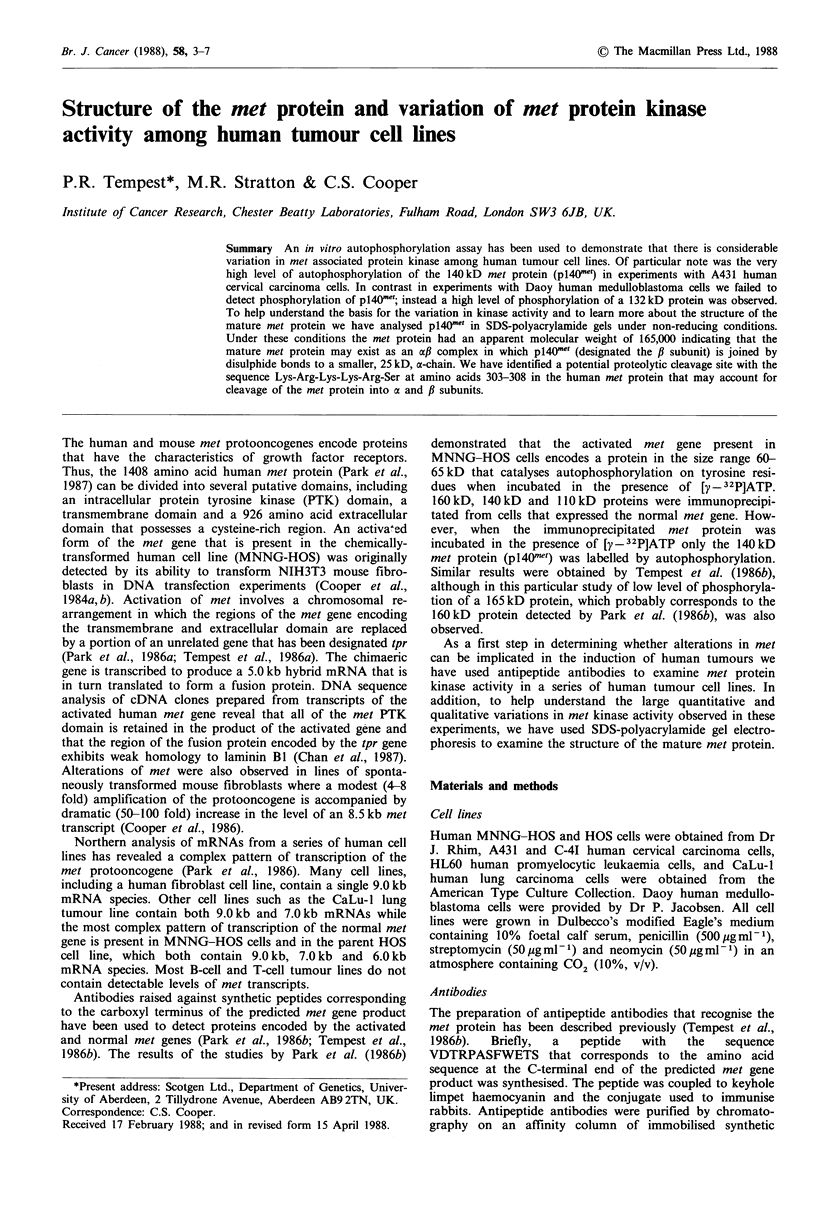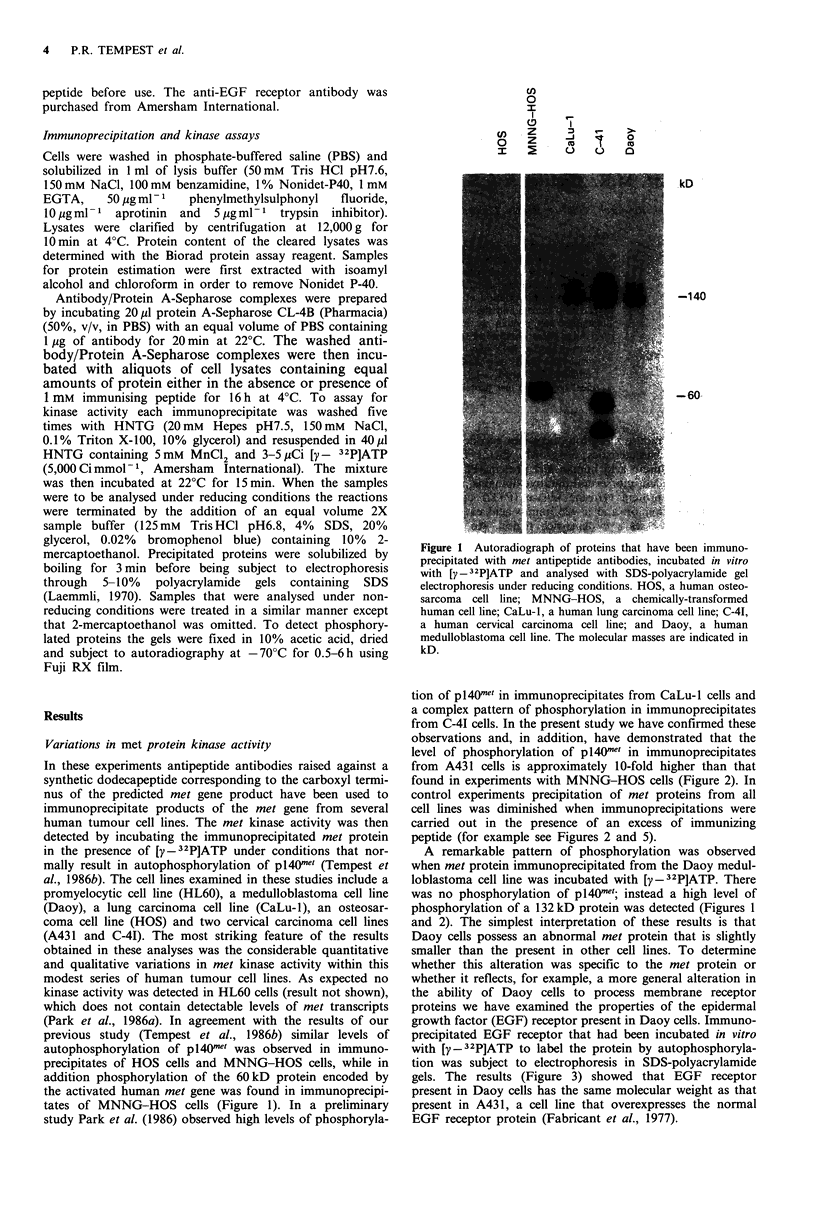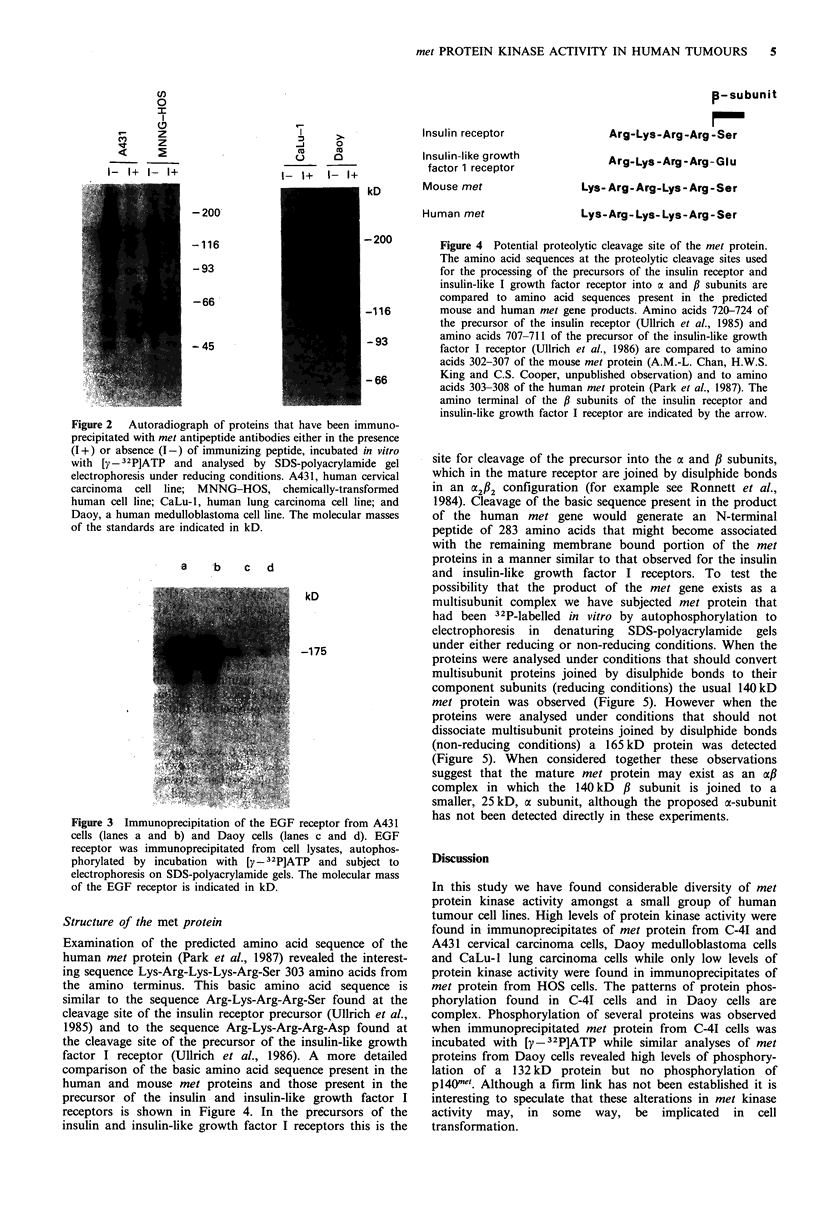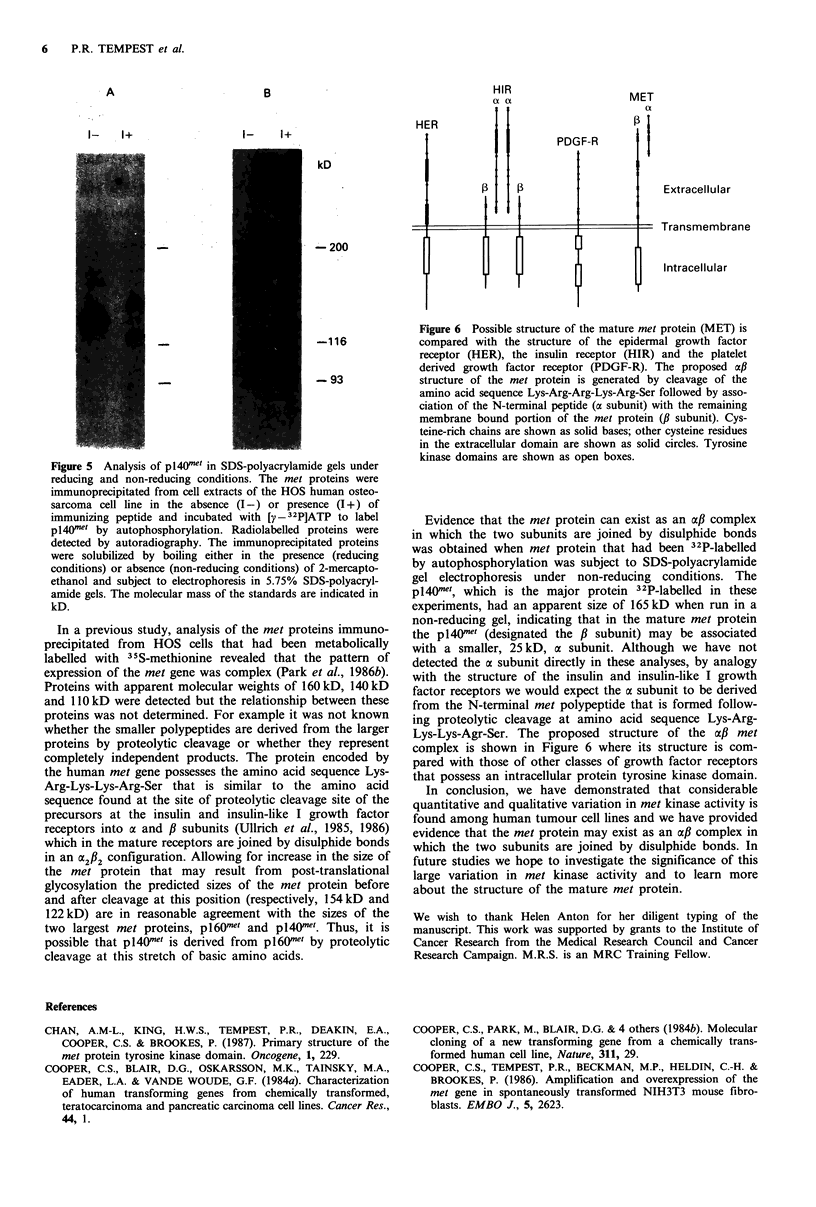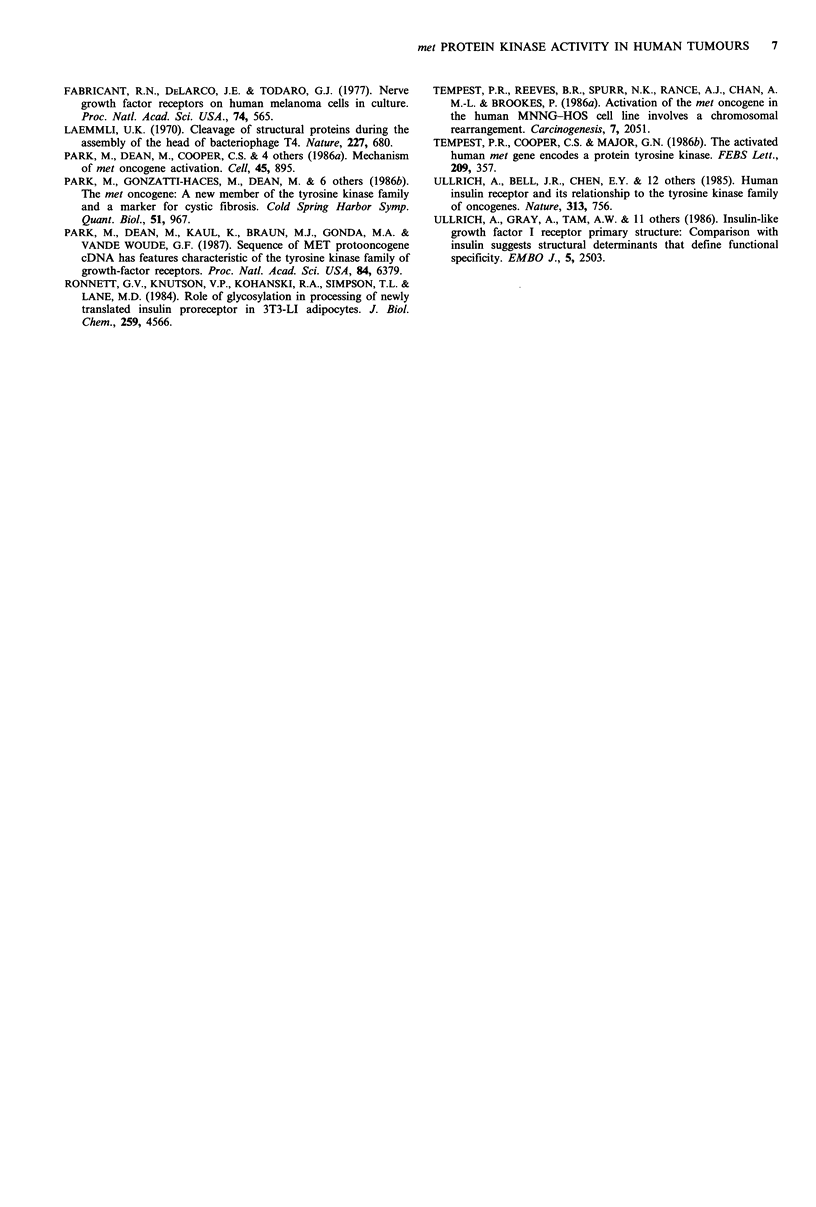# Structure of the met protein and variation of met protein kinase activity among human tumour cell lines.

**DOI:** 10.1038/bjc.1988.150

**Published:** 1988-07

**Authors:** P. R. Tempest, M. R. Stratton, C. S. Cooper

**Affiliations:** Institute of Cancer Research, Chester Beatty Laboratories, London, UK.

## Abstract

**Images:**


*Br. J. Cancer* (1988), **58**, 3–7 © The Macmillan Press Ltd., 1988

### Structure of the *met* protein and variation of *met* protein kinase activity among human tumour cell lines

P.R. Tempest*, M.R. Stratton & C.S. Cooper

*Institute of Cancer Research, Chester Beatty Laboratories, Fulham Road, London SW3 6JB, UK.*

**Summary** An *in vitro* autophosphorylation assay has been used to demonstrate that there is considerable variation in *met* associated protein kinase among human tumour cell lines. Of particular note was the very high level of autophosphorylation of the 140 kD *met* protein ($p140^{met}$) in experiments with A431 human cervical carcinoma cells. In contrast in experiments with Daoy human medulloblastoma cells we failed to detect phosphorylation of $p140^{met}$; instead a high level of phosphorylation of a 132 kD protein was observed. To help understand the basis for the variation in kinase activity and to learn more about the structure of the mature *met* protein we have analysed $p140^{met}$ in SDS-polyacrylamide gels under non-reducing conditions. Under these conditions the *met* protein had an apparent molecular weight of 165,000 indicating that the mature *met* protein may exist as an $\alpha\beta$ complex in which $p140^{met}$ (designated the $\beta$ subunit) is joined by disulphide bonds to a smaller, 25 kD, $\alpha$-chain. We have identified a potential proteolytic cleavage site with the sequence Lys-Arg-Lys-Lys-Arg-Ser at amino acids 303–308 in the human *met* protein that may account for cleavage of the *met* protein into $\alpha$ and $\beta$ subunits.

The human and mouse *met* protooncogenes encode proteins that have the characteristics of growth factor receptors. Thus, the 1408 amino acid human *met* protein (Park *et al.*, 1987) can be divided into several putative domains, including an intracellular protein tyrosine kinase (PTK) domain, a transmembrane domain and a 926 amino acid extracellular domain that possesses a cysteine-rich region. An activated form of the *met* gene that is present in the chemically-transformed human cell line (MNNG-HOS) was originally detected by its ability to transform NIH3T3 mouse fibroblasts in DNA transfection experiments (Cooper *et al.*, 1984*a*, *b*). Activation of *met* involves a chromosomal rearrangement in which the regions of the *met* gene encoding the transmembrane and extracellular domain are replaced by a portion of an unrelated gene that has been designated *tpr* (Park *et al.*, 1986*a*; Tempest *et al.*, 1986*a*). The chimaeric gene is transcribed to produce a 5.0 kb hybrid mRNA that is in turn translated to form a fusion protein. DNA sequence analysis of cDNA clones prepared from transcripts of the activated human *met* gene reveal that all of the *met* PTK domain is retained in the product of the activated gene and that the region of the fusion protein encoded by the *tpr* gene exhibits weak homology to laminin B1 (Chan *et al.*, 1987). Alterations of *met* were also observed in lines of spontaneously transformed mouse fibroblasts where a modest (4–8 fold) amplification of the protooncogene is accompanied by dramatic (50–100 fold) increase in the level of an 8.5 kb *met* transcript (Cooper *et al.*, 1986).

Northern analysis of mRNAs from a series of human cell lines has revealed a complex pattern of transcription of the *met* protooncogene (Park *et al.*, 1986). Many cell lines, including a human fibroblast cell line, contain a single 9.0 kb mRNA species. Other cell lines such as the CaLu-1 lung tumour line contain both 9.0 kb and 7.0 kb mRNAs while the most complex pattern of transcription of the normal *met* gene is present in MNNG–HOS cells and in the parent HOS cell line, which both contain 9.0 kb, 7.0 kb and 6.0 kb mRNA species. Most B-cell and T-cell tumour lines do not contain detectable levels of *met* transcripts.

Antibodies raised against synthetic peptides corresponding to the carboxyl terminus of the predicted *met* gene product have been used to detect proteins encoded by the activated and normal *met* genes (Park *et al.*, 1986*b*; Tempest *et al.*, 1986*b*). The results of the studies by Park *et al.* (1986*b*) demonstrated that the activated *met* gene present in MNNG–HOS cells encodes a protein in the size range 60–65 kD that catalyses autophosphorylation on tyrosine residues when incubated in the presence of $[\gamma-^{32}P]$ATP. 160 kD, 140 kD and 110 kD proteins were immunoprecipitated from cells that expressed the normal *met* gene. However, when the immunoprecipitated *met* protein was incubated in the presence of $[\gamma-^{32}P]$ATP only the 140 kD *met* protein ($p140^{met}$) was labelled by autophosphorylation. Similar results were obtained by Tempest *et al.* (1986*b*), although in this particular study of low level of phosphorylation of a 165 kD protein, which probably corresponds to the 160 kD protein detected by Park *et al.* (1986*b*), was also observed.

As a first step in determining whether alterations in *met* can be implicated in the induction of human tumours we have used antipeptide antibodies to examine *met* protein kinase activity in a series of human tumour cell lines. In addition, to help understand the large quantitative and qualitative variations in *met* kinase activity observed in these experiments, we have used SDS-polyacrylamide gel electrophoresis to examine the structure of the mature *met* protein.

### Materials and methods

### *Cell lines*

Human MNNG–HOS and HOS cells were obtained from Dr J. Rhim, A431 and C-4I human cervical carcinoma cells, HL60 human promyelocytic leukaemia cells, and CaLu-1 human lung carcinoma cells were obtained from the American Type Culture Collection. Daoy human medulloblastoma cells were provided by Dr P. Jacobsen. All cell lines were grown in Dulbecco's modified Eagle's medium containing 10% foetal calf serum, penicillin (500 $\mu g\,ml^{-1}$), streptomycin (50 $\mu g\,ml^{-1}$) and neomycin (50 $\mu g\,ml^{-1}$) in an atmosphere containing $CO_2$ (10%, v/v).

### *Antibodies*

The preparation of antipeptide antibodies that recognise the *met* protein has been described previously (Tempest *et al.*, 1986*b*). Briefly, a peptide with the sequence VDTRPASFWETS that corresponds to the amino acid sequence at the C-terminal end of the predicted *met* gene product was synthesised. The peptide was coupled to keyhole limpet haemocyanin and the conjugate used to immunise rabbits. Antipeptide antibodies were purified by chromatography on an affinity column of immobilised synthetic

*Present address: Scotgen Ltd., Department of Genetics, University of Aberdeen, 2 Tillydrone Avenue, Aberdeen AB9 2TN, UK. Correspondence: C.S. Cooper.
Received 17 February 1988; and in revised form 15 April 1988.

peptide before use. The anti-EGF receptor antibody was purchased from Amersham International.

*Immunoprecipitation and kinase assays*

Cells were washed in phosphate-buffered saline (PBS) and solubilized in 1 ml of lysis buffer (50 mM Tris HCl pH7.6, 150 mM NaCl, 100 mM benzamidine, 1% Nonidet-P40, 1 mM EGTA, 50 $\mu g\,ml^{-1}$ phenylmethylsulphonyl fluoride, 10 $\mu g\,ml^{-1}$ aprotinin and 5 $\mu g\,ml^{-1}$ trypsin inhibitor). Lysates were clarified by centrifugation at 12,000 g for 10 min at 4°C. Protein content of the cleared lysates was determined with the Biorad protein assay reagent. Samples for protein estimation were first extracted with isoamyl alcohol and chloroform in order to remove Nonidet P-40.

Antibody/Protein A-Sepharose complexes were prepared by incubating 20 $\mu$l protein A-Sepharose CL-4B (Pharmacia) (50%, v/v, in PBS) with an equal volume of PBS containing 1 $\mu$g of antibody for 20 min at 22°C. The washed antibody/Protein A-Sepharose complexes were then incubated with aliquots of cell lysates containing equal amounts of protein either in the absence or presence of 1 mM immunising peptide for 16 h at 4°C. To assay for kinase activity each immunoprecipitate was washed five times with HNTG (20 mM Hepes pH7.5, 150 mM NaCl, 0.1% Triton X-100, 10% glycerol) and resuspended in 40 $\mu$l HNTG containing 5 mM $MnCl_2$ and 3–5 $\mu$Ci $[\gamma-{}^{32}P]$ATP (5,000 Ci $mmol^{-1}$, Amersham International). The mixture was then incubated at 22°C for 15 min. When the samples were to be analysed under reducing conditions the reactions were terminated by the addition of an equal volume 2X sample buffer (125 mM TrisHCl pH6.8, 4% SDS, 20% glycerol, 0.02% bromophenol blue) containing 10% 2-mercaptoethanol. Precipitated proteins were solubilized by boiling for 3 min before being subject to electrophoresis through 5–10% polyacrylamide gels containing SDS (Laemmli, 1970). Samples that were analysed under non-reducing conditions were treated in a similar manner except that 2-mercaptoethanol was omitted. To detect phosphorylated proteins the gels were fixed in 10% acetic acid, dried and subject to autoradiography at −70°C for 0.5–6 h using Fuji RX film.

**Figure 1** Autoradiograph of proteins that have been immunoprecipitated with *met* antipeptide antibodies, incubated *in vitro* with $[\gamma-{}^{32}P]$ATP and analysed with SDS-polyacrylamide gel electrophoresis under reducing conditions. HOS, a human osteosarcoma cell line; MNNG–HOS, a chemically-transformed human cell line; CaLu-1, a human lung carcinoma cell line; C-4I, a human cervical carcinoma cell line; and Daoy, a human medulloblastoma cell line. The molecular masses are indicated in kD.

### Results

*Variations in* met *protein kinase activity*

In these experiments antipeptide antibodies raised against a synthetic dodecapeptide corresponding to the carboxyl terminus of the predicted *met* gene product have been used to immunoprecipitate products of the *met* gene from several human tumour cell lines. The *met* kinase activity was then detected by incubating the immunoprecipitated *met* protein in the presence of $[\gamma-{}^{32}P]$ATP under conditions that normally result in autophosphorylation of $p140^{met}$ (Tempest *et al.*, 1986*b*). The cell lines examined in these studies include a promyelocytic cell line (HL60), a medulloblastoma cell line (Daoy), a lung carcinoma cell line (CaLu-1), an osteosarcoma cell line (HOS) and two cervical carcinoma cell lines (A431 and C-4I). The most striking feature of the results obtained in these analyses was the considerable quantitative and qualitative variations in *met* kinase activity within this modest series of human tumour cell lines. As expected no kinase activity was detected in HL60 cells (result not shown), which does not contain detectable levels of *met* transcripts (Park *et al.*, 1986*a*). In agreement with the results of our previous study (Tempest *et al.*, 1986*b*) similar levels of autophosphorylation of $p140^{met}$ was observed in immunoprecipitates of HOS cells and MNNG–HOS cells, while in addition phosphorylation of the 60 kD protein encoded by the activated human *met* gene was found in immunoprecipitates of MNNG–HOS cells (Figure 1). In a preliminary study Park *et al.* (1986) observed high levels of phosphorylation of $p140^{met}$ in immunoprecipitates from CaLu-1 cells and a complex pattern of phosphorylation in immunoprecipitates from C-4I cells. In the present study we have confirmed these observations and, in addition, have demonstrated that the level of phosphorylation of $p140^{met}$ in immunoprecipitates from A431 cells is approximately 10-fold higher than that found in experiments with MNNG–HOS cells (Figure 2). In control experiments precipitation of *met* proteins from all cell lines was diminished when immunoprecipitations were carried out in the presence of an excess of immunizing peptide (for example see Figures 2 and 5).

A remarkable pattern of phosphorylation was observed when *met* protein immunoprecipitated from the Daoy medulloblastoma cell line was incubated with $[\gamma-{}^{32}P]$ATP. There was no phosphorylation of $p140^{met}$; instead a high level of phosphorylation of a 132 kD protein was detected (Figures 1 and 2). The simplest interpretation of these results is that Daoy cells possess an abnormal *met* protein that is slightly smaller than the present in other cell lines. To determine whether this alteration was specific to the *met* protein or whether it reflects, for example, a more general alteration in the ability of Daoy cells to process membrane receptor proteins we have examined the properties of the epidermal growth factor (EGF) receptor present in Daoy cells. Immunoprecipitated EGF receptor that had been incubated *in vitro* with $[\gamma-{}^{32}P]$ATP to label the protein by autophosphorylation was subject to electrophoresis in SDS-polyacrylamide gels. The results (Figure 3) showed that EGF receptor present in Daoy cells has the same molecular weight as that present in A431, a cell line that overexpresses the normal EGF receptor protein (Fabricant *et al.*, 1977).

**Figure 2** Autoradiograph of proteins that have been immunoprecipitated with *met* antipeptide antibodies either in the presence (I+) or absence (I−) of immunizing peptide, incubated *in vitro* with [$\gamma-{}^{32}$P]ATP and analysed by SDS-polyacrylamide gel electrophoresis under reducing conditions. A431, human cervical carcinoma cell line; MNNG–HOS, chemically-transformed human cell line; CaLu-1, human lung carcinoma cell line; and Daoy, a human medulloblastoma cell line. The molecular masses of the standards are indicated in kD.

**Figure 3** Immunoprecipitation of the EGF receptor from A431 cells (lanes a and b) and Daoy cells (lanes c and d). EGF receptor was immunoprecipitated from cell lysates, autophosphorylated by incubation with [$\gamma-{}^{32}$P]ATP and subject to electrophoresis on SDS-polyacrylamide gels. The molecular mass of the EGF receptor is indicated in kD.

### *Structure of the* met *protein*

Examination of the predicted amino acid sequence of the human *met* protein (Park *et al.*, 1987) revealed the interesting sequence Lys-Arg-Lys-Lys-Arg-Ser 303 amino acids from the amino terminus. This basic amino acid sequence is similar to the sequence Arg-Lys-Arg-Arg-Ser found at the cleavage site of the insulin receptor precursor (Ullrich *et al.*, 1985) and to the sequence Arg-Lys-Arg-Arg-Asp found at the cleavage site of the precursor of the insulin-like growth factor I receptor (Ullrich *et al.*, 1986). A more detailed comparison of the basic amino acid sequence present in the human and mouse *met* proteins and those present in the precursor of the insulin and insulin-like growth factor I receptors is shown in Figure 4. In the precursors of the insulin and insulin-like growth factor I receptors this is the site for cleavage of the precursor into the α and β subunits, which in the mature receptor are joined by disulphide bonds in an $\alpha_2\beta_2$ configuration (for example see Ronnett *et al.*, 1984). Cleavage of the basic sequence present in the product of the human *met* gene would generate an N-terminal peptide of 283 amino acids that might become associated with the remaining membrane bound portion of the *met* proteins in a manner similar to that observed for the insulin and insulin-like growth factor I receptors. To test the possibility that the product of the *met* gene exists as a multisubunit complex we have subjected *met* protein that had been $^{32}$P-labelled *in vitro* by autophosphorylation to electrophoresis in denaturing SDS-polyacrylamide gels under either reducing or non-reducing conditions. When the proteins were analysed under conditions that should convert multisubunit proteins joined by disulphide bonds to their component subunits (reducing conditions) the usual 140 kD *met* protein was observed (Figure 5). However when the proteins were analysed under conditions that should not dissociate multisubunit proteins joined by disulphide bonds (non-reducing conditions) a 165 kD protein was detected (Figure 5). When considered together these observations suggest that the mature *met* protein may exist as an αβ complex in which the 140 kD β subunit is joined to a smaller, 25 kD, α subunit, although the proposed α-subunit has not been detected directly in these experiments.

| | β-subunit |
|---|---|
| Insulin receptor | **Arg-Lys-Arg-Arg-Ser** |
| Insulin-like growth factor 1 receptor | **Arg-Lys-Arg-Arg-Glu** |
| Mouse *met* | **Lys-Arg-Arg-Lys-Arg-Ser** |
| Human *met* | **Lys-Arg-Lys-Lys-Arg-Ser** |

**Figure 4** Potential proteolytic cleavage site of the *met* protein. The amino acid sequences at the proteolytic cleavage sites used for the processing of the precursors of the insulin receptor and insulin-like I growth factor receptor into α and β subunits are compared to amino acid sequences present in the predicted mouse and human *met* gene products. Amino acids 720–724 of the precursor of the insulin receptor (Ullrich *et al.*, 1985) and amino acids 707–711 of the precursor of the insulin-like growth factor I receptor (Ullrich *et al.*, 1986) are compared to amino acids 302–307 of the mouse *met* protein (A.M.-L. Chan, H.W.S. King and C.S. Cooper, unpublished observation) and to amino acids 303–308 of the human *met* protein (Park *et al.*, 1987). The amino terminal of the β subunits of the insulin receptor and insulin-like growth factor I receptor are indicated by the arrow.

### Discussion

In this study we have found considerable diversity of *met* protein kinase activity amongst a small group of human tumour cell lines. High levels of protein kinase activity were found in immunoprecipitates of *met* protein from C-4I and A431 cervical carcinoma cells, Daoy medulloblastoma cells and CaLu-1 lung carcinoma cells while only low levels of protein kinase activity were found in immunoprecipitates of *met* protein from HOS cells. The patterns of protein phosphorylation found in C-4I cells and in Daoy cells are complex. Phosphorylation of several proteins was observed when immunoprecipitated *met* protein from C-4I cells was incubated with [$\gamma-{}^{32}$P]ATP while similar analyses of *met* proteins from Daoy cells revealed high levels of phosphorylation of a 132 kD protein but no phosphorylation of $p140^{met}$. Although a firm link has not been established it is interesting to speculate that these alterations in *met* kinase activity may, in some way, be implicated in cell transformation.

**Figure 5** Analysis of p140$^{met}$ in SDS-polyacrylamide gels under reducing and non-reducing conditions. The *met* proteins were immunoprecipitated from cell extracts of the HOS human osteosarcoma cell line in the absence (I−) or presence (I+) of immunizing peptide and incubated with [$\gamma-{}^{32}$P]ATP to label p140$^{met}$ by autophosphorylation. Radiolabelled proteins were detected by autoradiography. The immunoprecipitated proteins were solubilized by boiling either in the presence (reducing conditions) or absence (non-reducing conditions) of 2-mercapto-ethanol and subject to electrophoresis in 5.75% SDS-polyacrylamide gels. The molecular mass of the standards are indicated in kD.

**Figure 6** Possible structure of the mature *met* protein (MET) is compared with the structure of the epidermal growth factor receptor (HER), the insulin receptor (HIR) and the platelet derived growth factor receptor (PDGF-R). The proposed $\alpha\beta$ structure of the *met* protein is generated by cleavage of the amino acid sequence Lys-Arg-Arg-Lys-Arg-Ser followed by association of the N-terminal peptide ($\alpha$ subunit) with the remaining membrane bound portion of the *met* protein ($\beta$ subunit). Cysteine-rich chains are shown as solid bases; other cysteine residues in the extracellular domain are shown as solid circles. Tyrosine kinase domains are shown as open boxes.

In a previous study, analysis of the *met* proteins immunoprecipitated from HOS cells that had been metabolically labelled with $^{35}$S-methionine revealed that the pattern of expression of the *met* gene was complex (Park *et al.*, 1986*b*). Proteins with apparent molecular weights of 160 kD, 140 kD and 110 kD were detected but the relationship between these proteins was not determined. For example it was not known whether the smaller polypeptides are derived from the larger proteins by proteolytic cleavage or whether they represent completely independent products. The protein encoded by the human *met* gene possesses the amino acid sequence Lys-Arg-Lys-Lys-Arg-Ser that is similar to the amino acid sequence found at the site of proteolytic cleavage site of the precursors at the insulin and insulin-like I growth factor receptors into $\alpha$ and $\beta$ subunits (Ullrich *et al.*, 1985, 1986) which in the mature receptors are joined by disulphide bonds in an $\alpha_2\beta_2$ configuration. Allowing for increase in the size of the *met* protein that may result from post-translational glycosylation the predicted sizes of the *met* protein before and after cleavage at this position (respectively, 154 kD and 122 kD) are in reasonable agreement with the sizes of the two largest *met* proteins, p160$^{met}$ and p140$^{met}$. Thus, it is possible that p140$^{met}$ is derived from p160$^{met}$ by proteolytic cleavage at this stretch of basic amino acids.

Evidence that the *met* protein can exist as an $\alpha\beta$ complex in which the two subunits are joined by disulphide bonds was obtained when *met* protein that had been $^{32}$P-labelled by autophosphorylation was subject to SDS-polyacrylamide gel electrophoresis under non-reducing conditions. The p140$^{met}$, which is the major protein $^{32}$P-labelled in these experiments, had an apparent size of 165 kD when run in a non-reducing gel, indicating that in the mature *met* protein the p140$^{met}$ (designated the $\beta$ subunit) may be associated with a smaller, 25 kD, $\alpha$ subunit. Although we have not detected the $\alpha$ subunit directly in these analyses, by analogy with the structure of the insulin and insulin-like I growth factor receptors we would expect the $\alpha$ subunit to be derived from the N-terminal *met* polypeptide that is formed following proteolytic cleavage at amino acid sequence Lys-Arg-Lys-Lys-Agr-Ser. The proposed structure of the $\alpha\beta$ *met* complex is shown in Figure 6 where its structure is compared with those of other classes of growth factor receptors that possess an intracellular protein tyrosine kinase domain.

In conclusion, we have demonstrated that considerable quantitative and qualitative variation in *met* kinase activity is found among human tumour cell lines and we have provided evidence that the *met* protein may exist as an $\alpha\beta$ complex in which the two subunits are joined by disulphide bonds. In future studies we hope to investigate the significance of this large variation in *met* kinase activity and to learn more about the structure of the mature *met* protein.

We wish to thank Helen Anton for her diligent typing of the manuscript. This work was supported by grants to the Institute of Cancer Research from the Medical Research Council and Cancer Research Campaign. M.R.S. is an MRC Training Fellow.

### References

CHAN, A.M-L., KING, H.W.S., TEMPEST, P.R., DEAKIN, E.A., COOPER, C.S. & BROOKES, P. (1987). Primary structure of the *met* protein tyrosine kinase domain. *Oncogene*, **1**, 229.

COOPER, C.S., BLAIR, D.G., OSKARSSON, M.K., TAINSKY, M.A., EADER, L.A. & VANDE WOUDE, G.F. (1984*a*). Characterization of human transforming genes from chemically transformed, teratocarcinoma and pancreatic carcinoma cell lines. *Cancer Res.*, **44**, 1.

COOPER, C.S., PARK, M., BLAIR, D.G. & 4 others (1984*b*). Molecular cloning of a new transforming gene from a chemically transformed human cell line, *Nature*, **311**, 29.

COOPER, C.S., TEMPEST, P.R., BECKMAN, M.P., HELDIN, C.-H. & BROOKES, P. (1986). Amplification and overexpression of the *met* gene in spontaneously transformed NIH3T3 mouse fibroblasts. *EMBO J.*, **5**, 2623.

FABRICANT, R.N., DELARCO, J.E. & TODARO, G.J. (1977). Nerve growth factor receptors on human melanoma cells in culture. *Proc. Natl. Acad. Sci. USA.*, **74**, 565.

LAEMMLI, U.K. (1970). Cleavage of structural proteins during the assembly of the head of bacteriophage T4. *Nature*, **227**, 680.

PARK, M., DEAN, M., COOPER, C.S. & 4 others (1986*a*). Mechanism of *met* oncogene activation. *Cell*, **45**, 895.

PARK, M., GONZATTI-HACES, M., DEAN, M. & 6 others (1986*b*). The *met* oncogene: A new member of the tyrosine kinase family and a marker for cystic fibrosis. *Cold Spring Harbor Symp. Quant. Biol.*, **51**, 967.

PARK, M., DEAN, M., KAUL, K., BRAUN, M.J., GONDA, M.A. & VANDE WOUDE, G.F. (1987). Sequence of MET protooncogene cDNA has features characteristic of the tyrosine kinase family of growth-factor receptors. *Proc. Natl. Acad. Sci. USA*, **84**, 6379.

RONNETT, G.V., KNUTSON, V.P., KOHANSKI, R.A., SIMPSON, T.L. & LANE, M.D. (1984). Role of glycosylation in processing of newly translated insulin proreceptor in 3T3-LI adipocytes. *J. Biol. Chem.*, **259**, 4566.

TEMPEST, P.R., REEVES, B.R., SPURR, N.K., RANCE, A.J., CHAN, A. M.-L. & BROOKES, P. (1986*a*). Activation of the *met* oncogene in the human MNNG–HOS cell line involves a chromosomal rearrangement. *Carcinogenesis*, **7**, 2051.

TEMPEST, P.R., COOPER, C.S. & MAJOR, G.N. (1986*b*). The activated human *met* gene encodes a protein tyrosine kinase. *FEBS Lett.*, **209**, 357.

ULLRICH, A., BELL, J.R., CHEN, E.Y. & 12 others (1985). Human insulin receptor and its relationship to the tyrosine kinase family of oncogenes. *Nature*, **313**, 756.

ULLRICH, A., GRAY, A., TAM, A.W. & 11 others (1986). Insulin-like growth factor I receptor primary structure: Comparison with insulin suggests structural determinants that define functional specificity. *EMBO J.*, **5**, 2503.